# Driver or passenger effects of augmented *c-Myc* and *Cdc20* in gliomagenesis

**DOI:** 10.18632/oncotarget.8080

**Published:** 2016-03-14

**Authors:** Ping Ji, Xinhui Zhou, Qun Liu, Gregory N. Fuller, Lynette M. Phillips, Wei Zhang

**Affiliations:** ^1^ Department of Pathology, The University of Texas MD Anderson Cancer Center, Houston, Texas, USA; ^2^ Department of Neurosurgery, National Clinical Research Center for Cancer, Key Laboratory of Cancer Prevention and Therapy of Tianjin, Tianjin Medical University Cancer Institute and Hospital, Tianjin, PR China; ^3^ Current affiliation: Department of Biochemistry and Molecular Biology, The University of Texas Medical Branch, Galveston, Texas, USA

**Keywords:** Myc, Cdc20, RCAS/Ntv-a glia-specific mouse model, glial progenitor cell differentiation, glioblastoma

## Abstract

**Purpose:**

*Cdc20* and *c-Myc* are commonly overexpressed in a broad spectrum of cancers, including glioblastoma (GBM). Despite this clear association, whether *c-Myc* and *Cdc20* overexpression is a driver or passenger event in gliomagenesis remains unclear.

**Results:**

Both *c-Myc* and *Cdc20* induced the proliferation of primary glial progenitor cells. *c-Myc* also promoted the formation of soft agar anchorage-independent colonies. In the RCAS/*Ntv-a* glia-specific transgenic mouse model, *c-Myc* increased the GBM incidence from 19.1% to 47.4% by 12 weeks of age when combined with *kRas* and *Akt3* in *Ntv-a INK4a-ARF* (also known as *CDKN2A*)-null mice. In contrast, *Cdc20* decreased the GBM incidence from 19.1% to 9.1%. Moreover, cell differentiation was modulated by *c-Myc* in kRas/Akt3-induced GBM on the basis of Nestin/GFAP expression (glial progenitor cell differentiation), while *Cdc20* had no effect on primary glial progenitor cell differentiation.

**Materials and Methods:**

We used glial progenitor cells from Ntv-a newborn mice to evaluate the role of *c-Myc* and *Cdc20* in the proliferation and transformation of GBM *in vitro* and *in vivo*. We further determined whether *c-Myc* and *Cdc20* have a driver or passenger role in GBM development using *kRas/Akt3* signals in a RCAS/Ntv-a mouse model.

**Conclusions:**

These results suggest that the driver or passenger of oncogene signaling is dependent on cellular status. *c-Myc* is a driver when combined with *kRas/Akt3* oncogenic signals in gliomagenesis, whereas *Cdc20* overexpression is a passenger. Inhibition of cell differentiation of *c-Myc* may be a target for anti-glioma therapy.

## INTRODUCTION

Glioma represents approximately 70% of primary brain tumors in adults, with an incidence of about 6 cases per 100,000 individuals worldwide [1]. The most advanced form of glioma, as well as the most invasive and resistant to therapy, is glioblastoma (GBM), which comprises 50%–60% of all gliomas. These tumors arise from three different types of glial cells that are normally found in the brain: astrocytes, oligodendrocytes, and ependymal cells. There are a number of histological subtypes, as stratified by the World Health Organization (WHO). Grade I and II low-grade diffuse gliomas are slow-growing, less aggressive tumors, while grade III and IV are more aggressive malignant tumors. The most common form, with the worst prognosis, is GBM (WHO grade IV). Patients with GBM usually survive less than 15 months following diagnosis. Currently, there are no effective long-term treatments for this disease [2, 3].

Recent research by The Cancer Genome Atlas (TCGA), together with intensive research over the last three decades, has revealed a large number of putative oncogenes that are amplified or overexpressed in glioma [4–6], including c-*Myc* and *Cdc20*. C-Myc is a well-known transcription factor with roles in cell cycle progression, apoptosis, and longevity during development [7] and oncogenesis [8] and in stem cell self-renewal, differentiation, and metabolic reprogramming [9, 10]. Elevated expression of c-*Myc* is associated with aggressive disease and a poor clinical outcome [11, 12]. It is also notable that both the p53 and PTEN/PI3K pathways can directly regulate c-Myc, with p53 repressing c-*Myc* transcription by directly binding to the c-*Myc* promoter, whereas downstream PI3K pathway arms can modulate c-*Myc* translation and protein degradation [13]. Cdc20 appears to act as a regulatory protein that interacts with several other proteins at multiple points in the cell cycle [14–16]. It is required for two microtubule-dependent processes, nuclear movement prior to anaphase and chromosome separation [15, 17]. *Cdc20* is highly expressed in many types of cancer, including GBM [17, 18]. Recently, studies have shown that *Cdc20* maintains tumor initiation cells and serves as a potential target for cancer treatment [18–21]. However, wether *c-Myc* and *Cdc20* overexpression is a driver or passenger event in gliomagenesis remains unclear.

In this study, we used a powerful glia-specific mouse model (the RCAS/*Ntv-a* system) to characterize the oncogenic function of c-*Myc* and *Cdc20* in gliomagenesis *in vitro* and *in vivo*. Then, because single *c-Myc* and *Cdc20* genes are not sufficient to induce glioma in both CDKN2A+/+ and CDKN2A−/− Ntv-a mice, we explored the role of c-*Myc* and *Cdc20* in glioma development in a RCAS/Ntv-a mouse model using the combination of *kRas* and *Akt3*.

## RESULTS

### *C-Myc* and *Cdc20* promote glial progenitor cell proliferation and immortalization

**Figure 1:**
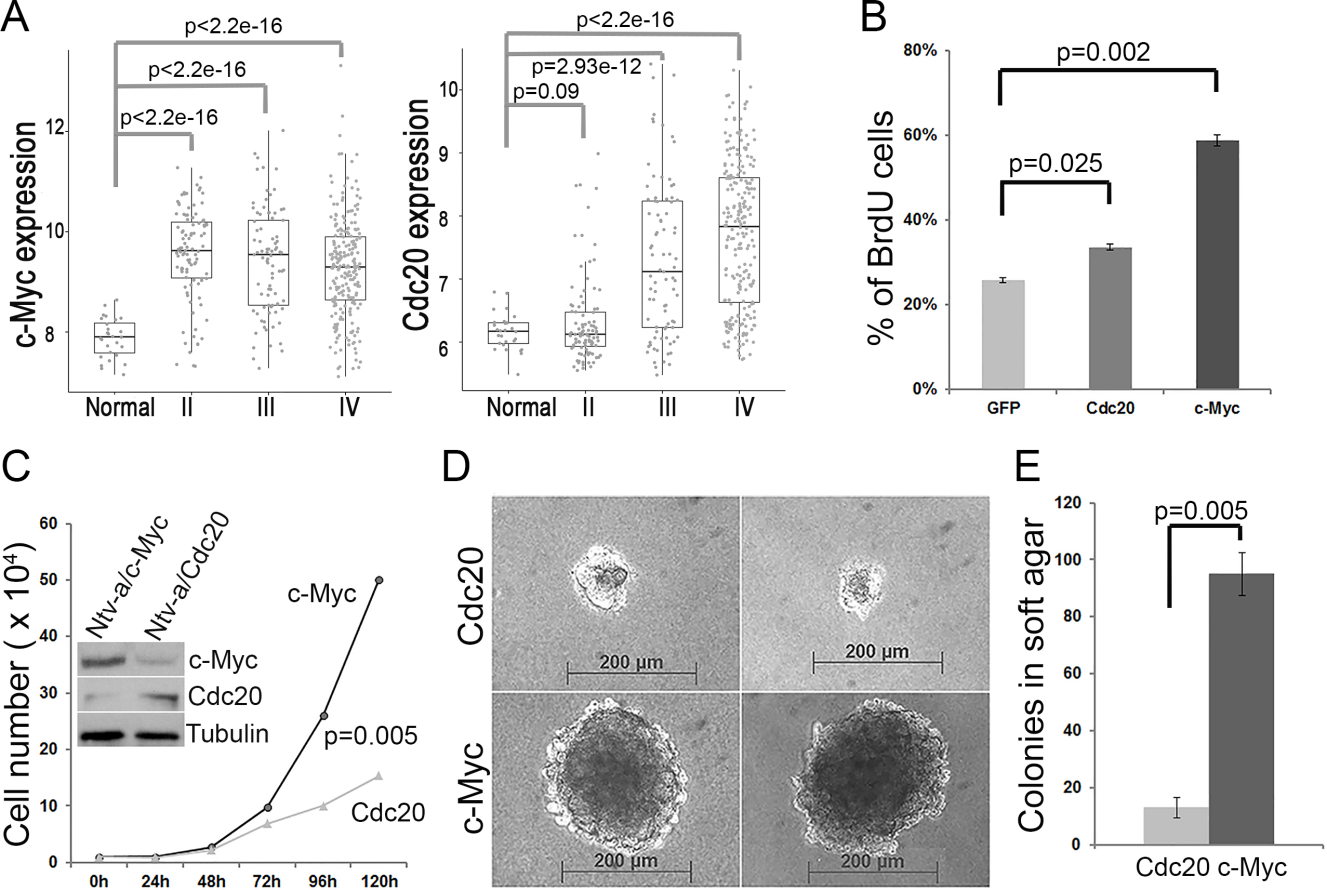
*c-Myc* and *Cdc20* promote glial progenitor cell proliferation and transformation (**A**) Expression of *Cdc20* and *c-Myc* was induced in GBM. Expression of *Cdc20* and *c-Myc* was analyzed in the patient tissue samples from the Rembrandt database, including normal tissues (28 cases), grade II glioma (99 cases), grade III glioma (85 cases), and grade IV GBM (228 cases). Expression of c-*Myc* and Cdc20 (signal intensity) was significantly different between normal tissues and grade IV GBM cases (*P* < 2.2e-16). (**B**) Glial progenitor cells were isolated from the brains of *Ntv-a* newborn mice and infected with RCAS-c-*Myc*, RCAS-*Cdc20*, or RCAS-*GFP*. The transduced cells were incubated with 10 μM BrdU for 1 hour and fixed with 70% ethanol. BrdU-positive cells were measured by flow cytometry after the cells were labeled with anti-BrdU antibody conjugated with FITC. The data were obtained from three replicates (*p* values, Student's *t*-test). (**C**) The immortalized cells with c-*Myc* or *Cdc20* were seeded in a 24-well plate at 1 × 10^4^ cells/well. The cell growth curve was drawn according to the mean number of cells, in triplicate, at the indicated time points. (**D**) The immortalized cells with c-*Myc* or *Cdc20* were seeded on a six-well plate with soft agar and incubated for 2 more weeks in cell culture conditions. A representative image of the colonies is shown. (**E**) The quantification of colonies was from three independent experiments.

We previously reported that some GBM signature genes, including c-*Myc* and *Cdc20*, distinguish GBM from other low-grade gliomas [22]. We analyzed the expression of these genes in patient tissue samples from the Rembrandt dataset and confirmed that they were both highly expressed in high-grade GBM samples compared to in normal control samples; Cdc20 expression was induced gradiently through low grade to high grade (Figure 1A). In addition, the expression of *c-Myc* and *Cdc20* was significantly higher in the samples with GBM in the TCGA GBM dataset than in the normal control samples (data not shown). To understand the oncogenic function of c-*Myc* and *Cdc20*, we performed proliferation and transformation assays *ex vivo* using primary glial progenitor cells from *Ntv-a* mice. As shown in Figure 1B, overexpression of c-*Myc* and *Cdc20* promoted glial progenitor cell proliferation (Figure 1B). Interestingly, the transduced glial progenitor cells with c-*Myc* or *Cdc20* could be cultured over 6 months, whereas *GFP* control cells were arrested in growth within 1 month. Moreover, the glial progenitor cells immortalized by c-*Myc* overexpression not only showed higher proliferation rates than did those immortalized by *Cdc20* overexpression (Figure 1C) but also gained the ability to form anchorage-independent colonies, unlike Cdc20-transduced cells (Figure 1D and 1E). C-*Myc* has stronger oncogenic activity than does *Cdc20* in promoting glial progenitor cell transformation.

### Oncogenic signal of c-*Myc* or *Cdc20* is insufficient to induce glioma, and the combination of *kRas* and *Akt3* is sufficient to induce GBM in *Ntv-a CDKN2A*-null mice

**Figure 2:**
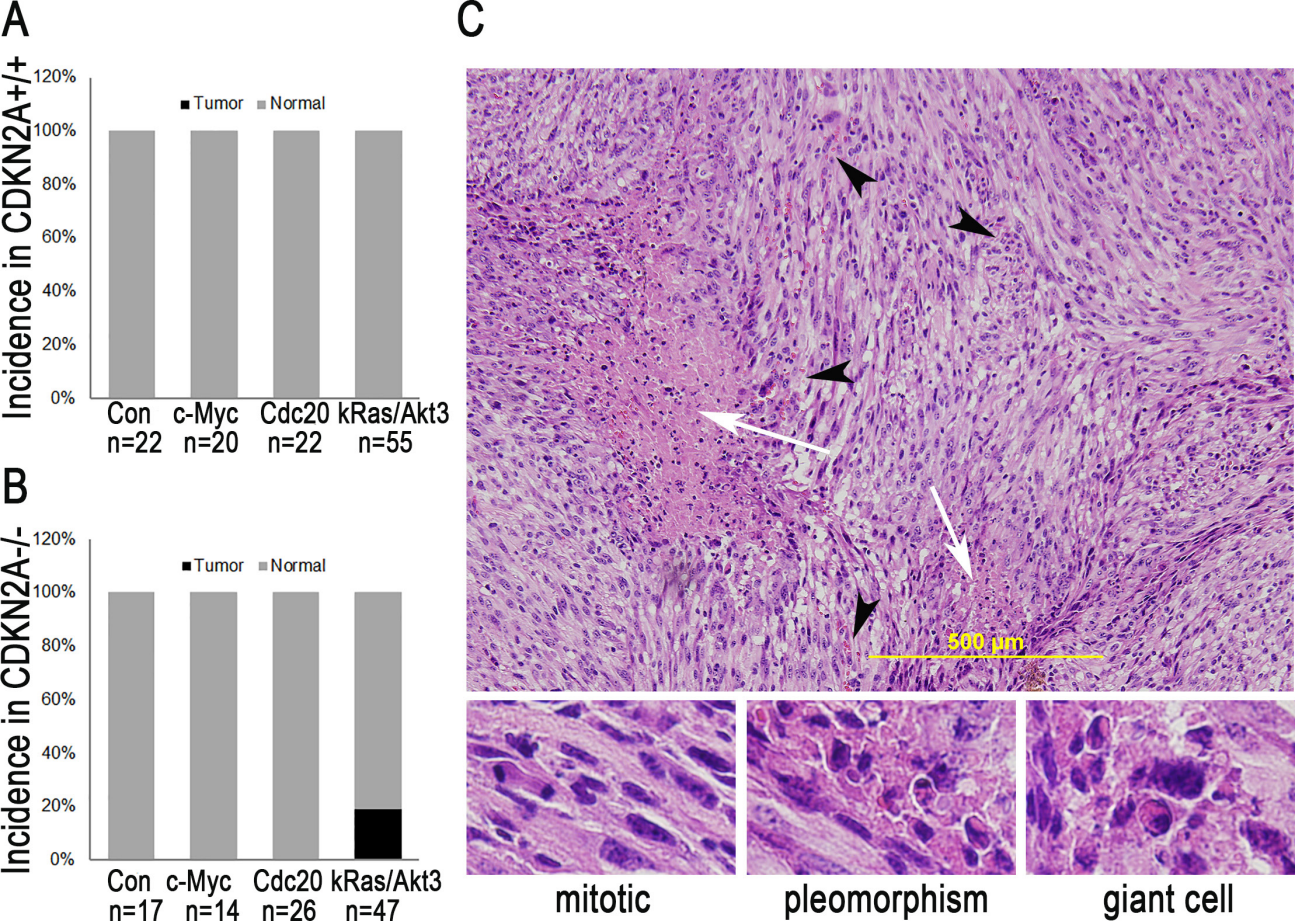
*c-Myc* or *Cdc20* is insufficient to induce glioma, but *kRas/Akt3* is sufficient to induce GBM in *Ntv-a CDKN2A*-null mice (**A**) c-*Myc* or *Cdc20* is insufficient to induce glioma in *Ntv-a CDKN2A* mice. (**B**) *kRas*/*Akt3* is sufficient to induce GBM in *Ntv-a CDKN2A*-null mice. (**C**) H & E staining of one representative tumor from *Ntv-a CDKN2A*-null mice injected with *kRas*/*Akt3* shows features of GBM, with palisading around necrotic foci (white arrow), glomeruloid vascular proliferation (black arrowhead) (up panel), and other features, such as mitotic cells, pleomorphism, and giant cells (low panel).

To investigate the causal role of c-*Myc* and *Cdc20* in glioma development, we injected *Ntv-a* transgenic mice with DF1 cells that produced RCAS-c-*Myc* or RCAS-*Cdc20*. A histopathologic examination of the brains from injected mice at the end of the 12-week study indicated that delivery of RCAS-c-*Myc* or RCAS-*Cdc20* alone failed to induce glioma (Figure 2A). Therefore, we built a glioma mouse model by combining *kRas* and *Akt* family genes in the RCAS/*Ntv-a* glia-specific mouse model. An analysis of GBM samples from TCGA revealed that the Akt signaling pathway is one of the most altered pathways in this tumor. *Akt3* is one of three closely related serine/threonine-protein kinases in the Akt family (*Akt1*, *Akt2*, and *Akt3*). In more recent years, it has become evident that *Akt3* is more important than is *Akt1* in glioma development and progression [23, 24]. Thus, we injected *Ntv-a* transgenic mice with DF1 cells that produced RCAS-*kRas* and RCAS-*Akt3*. As shown in Figure 2A, at the end of week 12, no gliomas had developed in 55 injected *Ntv-a* mice in two independent experiments. Because human high-grade glioma has additional deletions and mutations, such as loss of *CDKN2A*, which results in the disruption of cell cycle arrest pathways [25, 26], we further determined whether loss of *CDKN2A* is required for *kRas*- and *Akt3*-induced glioma by injecting *CDKN2A*-null *Ntv-a* transgenic mice with RCAS-*kRas* and RCAS-*Akt3*. Nine of 47 (19.1%) mice developed glioma within 12 weeks (Figure 2B). All of these tumors were GBM according to the histopathological criteria (microvascular proliferation and necrosis); other features included the appearance of mitotic cells, spindle cells, and giant cells (Figure 2C), as described in the WHO 2000 classification system. Therefore, loss of *CDKN2A* is required for GBM induction by *kRas* and *Akt3*.

### Combination of c-*Myc* and *kRas*/*Akt3*, but not *Cdc20*, enhances GBM development

**Figure 3:**
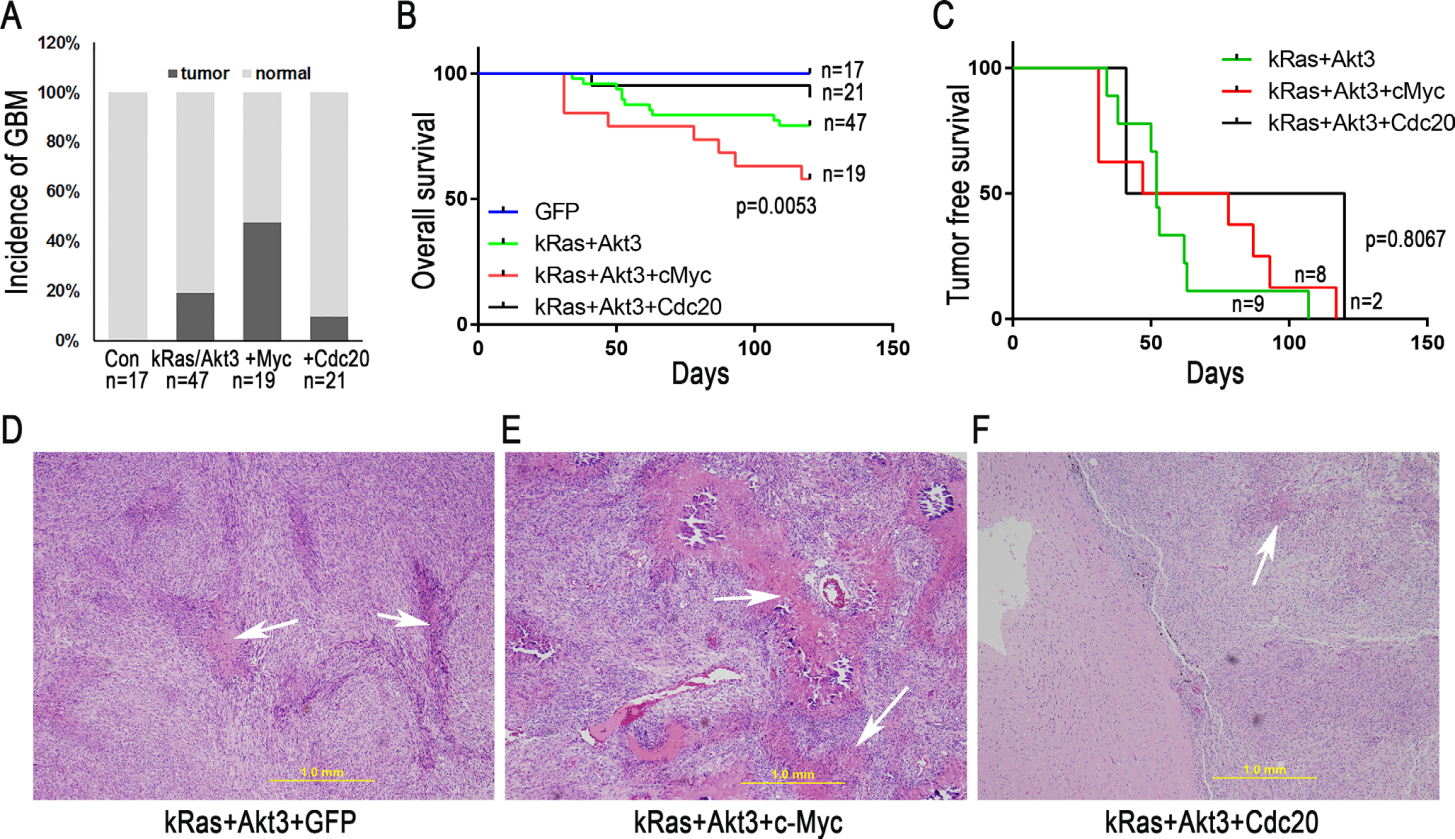
*c-Myc* promotes GBM development in *CDKN2A*-null mice with a combination of kRas and Akt3 signals (**A**) GBM incidence was increased by c-*Myc* combined with *kRas/Akt3* signals in *Ntv-a CDNK2A*-null mice. (**B**) Overall survival curve indicates the percentage of mice developing symptomatic tumors over time; the combination of c-*Myc* and *kRas*/*Akt3* resulted in poor survival (*p* = 0.0053, log-rank test for trend). (**C**) Tumor-free survival curve indicates that mice with *kRas*/*Akt3* and *kRas*/*Akt3*/c-*Myc* tumors had similar poor survival (*p* = 0.8067, log rank test for trend). (**D**–**F**) H & E staining of one representative tumor from each combination group with GBM features (microvascular proliferation and necrosis).

To determine the role of c-*Myc* and *Cdc20* in GBM development, we combined c-*Myc* or *Cdc20* signaling with *kRas*/*Akt3* oncogenic signals in the established mouse model. RCAS-c-*Myc* or RCAS-*Cdc20*, in combination with RCAS-*kRas* and RCAS-*Akt3*, were injected into *CDKN2A*-null *Ntv-a* mice. Nine of 19 mice (47.4%) with the c-*Myc*/*kRas*/*Akt3* combination developed GBM, with microvascular proliferation and necrosis (Figure 3A). In contrast, only 2 of 21 mice with the *Cdc20*/*kRas*/*Akt3* combination developed GBM (Figure 3A). Consistent with this, the overall survival duration was poor in mice injected with the c-*Myc*/*kRas*/*Akt3* combination (Figure 3B). However, tumor-free survival did not significantly differ among the *kRas*/*Akt3*, *kRas/Akt3/Cdc20*, and *kRas*/*Akt3*/c-*Myc* mice (Figure 3C). A histopathologic examination of the brain tumors indicated that they were GBM with microvascular proliferation and necrosis (Figure 3D–3F).

### Effect of c-*Myc* and *kRas*/*Akt3* on GBM development, with inhibition of glial progenitor cell differentiation

**Figure 4:**
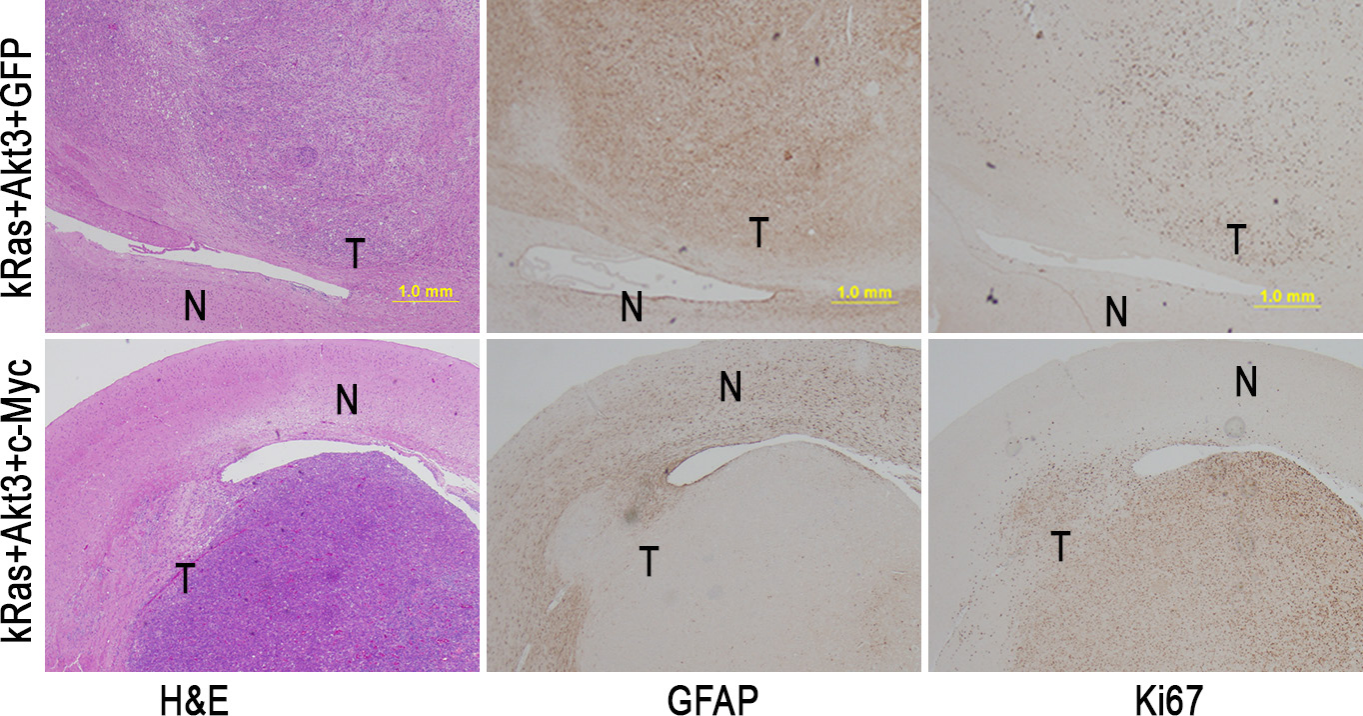
*c-Myc* promotes tumor cell proliferation with inhibition of cell differentiation A representative immunohistochemical staining image was shown in the tumors from *kRas*/*Akt3*/c-Myc and *kRas*/*Akt3/GFP* control tumors with indicated markers for Ki67 and GFAP. N, normal cells; T, tumor cells; scale bar, 1.0 mm.

**Figure 5:**
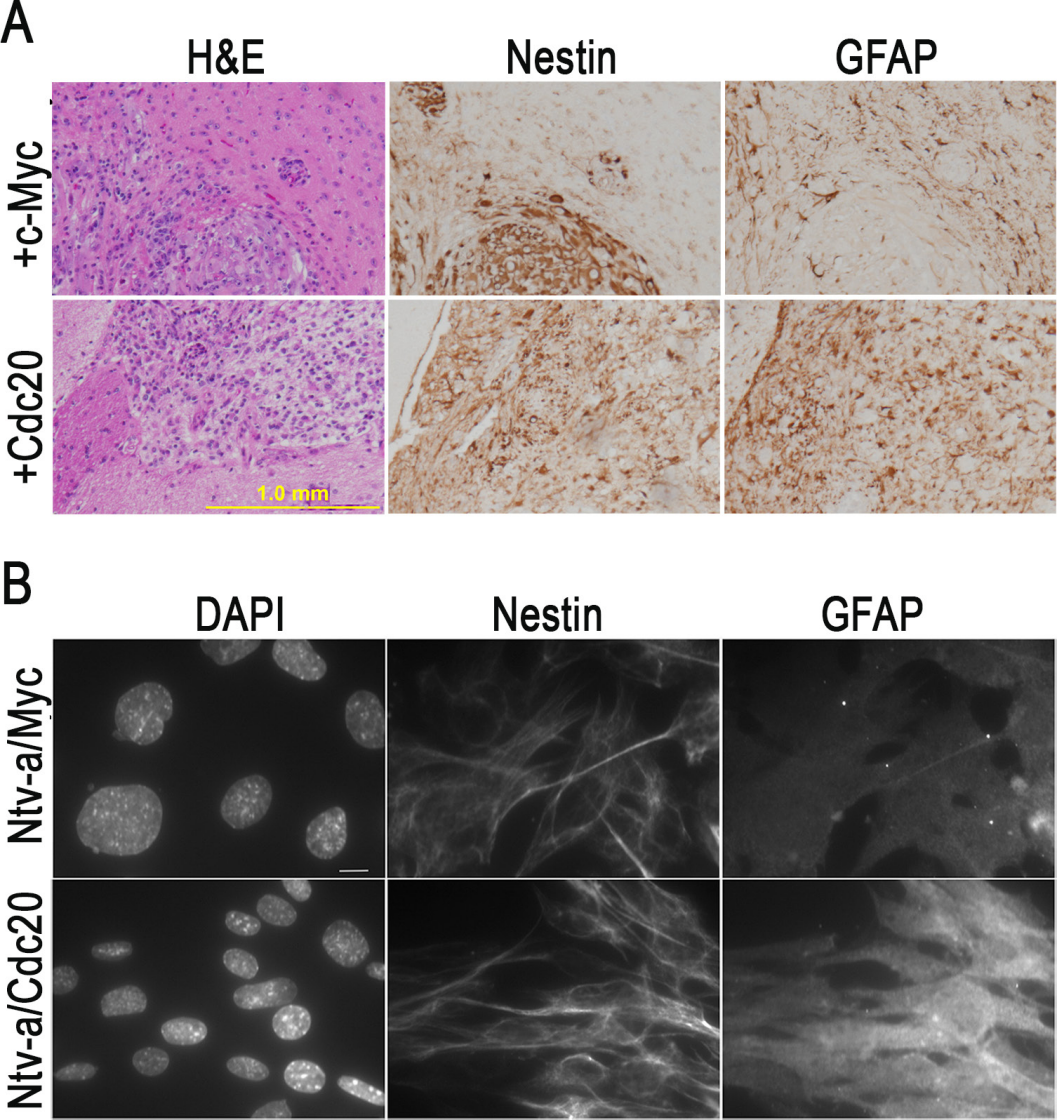
*c-Myc* inhibits glial progenitor cell differentiation *in vivo* and *in vitro* (**A**) Immunohistochemical staining of two representative *kRas*/*Akt3/c-Myc* and *Cdc20*/*kRas*/*Akt3* control tumors with indicated markers for Nestin and GFAP. (**B**) Immunofluorescence staining with GFAP and Nestin antibodies in c-Myc- or Cdc20-transformed glial progenitor cells. DAPI co-staining for genomic DNA, scale bar, 10 μm.

The experiments showed that c-*Myc* promotes GBM development when combined with *kRas* and *Akt3* signals in *CDKN2A*-null *Ntv-a* transgenic mice. The mouse GBMs were marked by high cellularity, prominent vascularity, and necrosis that were identical to those seen in human GBM. To further characterize the mouse GBM, we performed immunohistochemical staining with antibodies specific for glial cell differentiation marker GFAP and cell proliferation marker Ki67 and found that *c-Myc* inhibited cell differentiation and promoted cell proliferation (Figure 4). To determine whether the inhibition of tumor cell differentiation is unique in *c-Myc*-induced GBM, we performed immunohistochemical staining with antibodies that were specific for glial cell differentiation markers containing GFAP and Nestin and found that the resultant tumor cells expressed both Nestin and GFAP in *kRas/Akt3/c-Myc*- and *kRas*/*Akt3/Cdc20*-induced GBM (Figure 5A). However, GFAP levels were dramatically decreased in the tumor cells from c-*Myc*/*kRas*/*Akt3* combination mice. We also observed that GFAP expression was inhibited in c-*Myc*-transformed glial progenitor cells, while Cdc20 showed no effect in *Cdc20*-transformed cells (Figure 5B).

## DISCUSSION

Glioma represents the most common type of primary brain tumor. GBM, the most advanced form of glioma, is the most invasive and resistant to therapy. Intensive research over the last three decades, especially recent research by TCGA, has revealed a large number of putative oncogenes and tumor suppressor genes that are altered in glioma, such as *EGFR*, *PDGFRα*, *IGFBP2*, c-*Myc*, *Cdc20*, and *Akt3* oncogenes and *CDKN2A*, *PTEN*, and *NF1* tumor suppressor genes. However, many of these signature genes are only markers in a subgroup of glioma cases; their causal role in gliomagenesis remains unexplored. In this study, we used the glia-specific RCAS/*Ntv-a* somatic gene transfer mouse model, which has been used extensively to test oncogenes, singly [27, 28] and in combination [29, 30], for their ability to induce gliomagenesis. We provided definitive evidence that c-*Myc* and *Cdc20* alone are insufficient to induce glioma. Interestingly, glioma induction with the *kRas* and *Akt3* combination required *CDKN2A* deletion. Moreover, the combination of the oncogenes c-*Myc* or *Cdc20* in the context had distinct effects on the promotion and inhibition of GBM development.

We and others have shown that c-*Myc* and *Cdc20* are both highly expressed in GBM samples compared to in normal control samples. To understand the oncogenic function of c-*Myc* and *Cdc20*, we first performed cell proliferation assays *ex vivo* using primary glial progenitor cells from *Ntv-a* mice. Overexpression of c-*Myc* and *Cdc20* promoted glial progenitor cell proliferation. Moreover, the transduced glial progenitor cells with c-*Myc* or *Cdc20* could be cultured over 6 months, whereas *GFP* control cells were arrested in growth within 1 month. However, the glial progenitor cells immortalized by c-*Myc* overexpression not only showed higher proliferation rates than did those immortalized by *Cdc20* overexpression but also gained the ability to form anchorage-independent colonies, unlike Cdc20-transduced cells. This suggests that c-*Myc* has stronger oncogenic activity than does *Cdc20* in promoting glial progenitor cell transformation.

To investigate the causal role of c-*Myc* and *Cdc20* in glioma development, we induced *c-Myc* and *Cdc20* expression in our RCAS/*Ntv-a* glia-specific mouse model. The data indicated that delivery of RCAS-c-*Myc* or RCAS-*Cdc20* alone failed to induce glioma. Akt is among the most hyperactivated signaling pathways in human cancer and regulates key cellular functions, including cell growth, proliferation, angiogenesis, glucose metabolism, invasion, and survival. An analysis of human GBMs from TCGA revealed that the Akt signaling pathway is one of the top altered pathways in GBM [4, 5]. In addition, researchers have observed that Akt activation status is correlated with glioma grade [31]. The Akt family comprises three members (*Akt1*, *Akt2*, and *Akt3*) that share significant overall homological and kinase domain similarities but diverge in the linker region and the PH domain; they play distinct roles in development and tumor formation [23, 24, 32–35]. *Akt1* is the most well characterized isoform of the three, but in recent years, it has become evident that *Akt2* and *Akt3* are more important than is *Akt1* in glioma development and progression [23, 24, 36]. In this study, we used the well-characterized RCAS/*Ntv-a* glia-specific mouse model system to determine whether a combination of oncogenic signals induces GBM formation. First, we delivered RCAS-*kRas* and RCAS-*Akt3* to *Ntv-a* mice; no glioma developed within the 12-week study period. *CDNK2A* deletion is common in human high-grade glioma, and we observed that additional *CDKN2A* loss in the mouse model was required for the induction of GBM by the combination of *kRas* and *Akt3*. However, the combination of *kRas* and *Akt1* signals was sufficient to induce GBM in *Ntv-a* mice [29, 30]. These findings provide additional evidence that *Akt1* and *Akt3* play different roles in GBM development, dependent on their cellular location, interacting partners, and the context of other oncogenic signals.

Cancer development and progression involve the activation of multiple oncogenic signals. Identifying oncogenic drivers in glioma development and progression is a promising approach for targeted therapy. Oncogenic drivers can cause normal cells to become cancerous and promote the growth of tumors. They can be used to develop targeted therapies aimed at shutting down the signaling pathways. In this study, *c-Myc* not only induced primary glial progenitor cell proliferation, immortalization, and transformation but also promoted GBM formation (19.1% versus 47.7%) when combined with *kRas*/*Akt3* signals in our glia-specific mouse model. Although *Cdc20* induced primary glial progenitor cell proliferation and immortalization similar to *c-Myc*, it inhibited GBM formation (19.1% versus 9.1%) when combined with *kRas*/*Akt3* signals. On the basis of these data, we conclude that c-*Myc* serves as an oncogenic driver and may be a promising target for GBM treatment; this is consistent with the results of a previous report [37]; however, *Cdc20* is a passenger in GBM development and progression when combined with *kRas/Akt3* oncogenic signals.

## MATERIALS AND METHODS

### RCAS constructs and RCAS propagation in DF1 cells

RCAS-c-*Myc* and RCAS-*Cdc20* were constructed in our laboratory and have been validated by DNA sequencing and protein expression in DF1 cells. RCAS-*kRas* encodes full-length human mutant G12D-activated *kRas*. RCAS-*Akt3* carries the full length of human *Akt3* genes with the HA tag, kindly provided by Dr. Peter Vogt (The Scripps Research Institute La Jolla, CA 92037, USA). RCAS-*GFP*, containing full-length *GFP*, was used as an infection marker and was kindly provided by Yi Li (Baylor College of Medicine, Houston, TX). DF1 immortalized chicken fibroblasts were grown in DMEM with 10% FCS in a humidified incubator containing 10% CO_2_ at 39°C.

### Primary glial progenitor cell culture

All animal experiments were performed in accordance with The University of Texas MD Anderson Cancer Center (Houston, TX) Institutional Animal Care and Use Committee guidelines. Six newborn *Ntv-a* transgenic mice were killed on day one; their whole brains were mechanically cut into small pieces, washed with 1 × PBS (pH 7.4), and digested with 3 ml of 0.25% trypsin/1 mM EDTA in HBSS in sterile tubes that were then incubated in a 37°C water bath for 15 min, with gentle shaking every 5 min. After incubation, fresh DMEM/F12 with 10% FCS was added to terminate digestion. The supernatant containing primary cells was pelleted, washed once with fresh medium, and plated into 10-cm dishes for primary culture.

### *Ex vivo* infection of glial progenitor cells, cell proliferation, and soft agar assays

The supernatants containing various RCAS virus particles from DF-1 cell cultures were collected and filtered through 0.22-μm filters before being transferred into 60% confluent primary glial progenitor cells from *Ntv-a* newborn mice. The cells were infected once per day for 2 days. They were then seeded into three six-well plates overnight and incubated with 20 μM BrdU for 1 hour before being harvested, fixed with ethanol, stained with FITC-conjugated anti-BrdU antibody, and incubated with 7-AAD. Cell proliferation was measured by flow cytometry on the basis of BrdU-positive cells. For the immortalization assay, the transduced cells continued to pass for up to 6 months; 1 × 10^4^ cells were then seeded into 24-well plates, and cell growth was measured by counting the cell number. To determine whether *c-Myc* or *Cdc20* promotes colony formation in soft agar, the log phase cells were plated into soft agar plates, as described previously [38]. The plates were incubated for 2 weeks, and the colonies were counted under a microscope. The morphological features of the colony were captured using AxioVision3.1 microscopy.

### Injection of *Ntv-a* transgenic mice with RCAS viral particles

Both *Ntv-a CDNK2A*+/+ and *CDNK2A*−/− mice express *tv-a*, a receptor of RCAS virus that is modulated by glia-specific Nestin promoter; this virus has been previously described [27]. The mice were of mixed genetic background, including C57BL, 129, Balb/C, and FVB/N. RCAS viral particles were injected into *Ntv-a* transgenic mice, as described by Marucci et al. [17]. In brief, newborn mice (postnatal days 1–3) were intracranially injected (in the right frontal region) with 1 × 10^4^ DF1 cells producing RCAS virus alone or a combination using a 10-μl gas-tight Hamilton syringe. All mice were monitored daily and killed at the end of week 12, or earlier if they showed signs or symptoms of severe sickness or lethargy. The whole brains were collected and fixed in 4% formaldehyde in PBS for at least 24 h. Sections were analyzed by standard hematoxylin and eosin (H & E) staining.

### Brain sectioning and immunohistochemistry

Each brain was coronally sectioned into five 5-μm-thick slices with a Leica microtome (Bannockburn, IL). Immunostaining was performed with LSAB2 kits (DAKO Cytomation, Carpinteria, CA). In brief, deparaffinized slides were treated with antigen-unmasking reagent (DAKO) by being heated in a microwave steamer for 16 min, and sections were blocked with 0.03% peroxidase for 10 min at ambient temperature. Antibodies against Nestin and GFAP were purchased from Abcom, Inc. (Cambridge, MA) and Millipore (Billerica, MA). Primary antibodies were diluted (1:500–1000) in TBST and incubated with sections at 4°C overnight. After being washed with TBST, sections were incubated with secondary antibodies (DAKO) at ambient temperature for 30 min. Sections were again washed with TBST and developed after the addition of DAB mixture (DAKO). After the reaction was terminated, sections were counterstained with freshly filtered hematoxylin and mounted.
